# ERas Enhances Resistance to Cisplatin-Induced Apoptosis by Suppressing Autophagy in Gastric Cancer Cell

**DOI:** 10.3389/fcell.2019.00375

**Published:** 2020-01-21

**Authors:** Huajian Tian, Wenjun Wang, Xiao Meng, Miaomiao Wang, Junyang Tan, Wenjuan Jia, Peining Li, Jianshuang Li, Qinghua Zhou

**Affiliations:** ^1^The First Affiliated Hospital, Biomedical Translational Research Institute, Jinan University, Guangzhou, China; ^2^Qingyuan People’s Hospital, The Sixth Affiliated Hospital of Guangzhou Medical University, Qingyuan, China; ^3^Department of Genetics, Yale School of Medicine, Yale University, New Haven, CT, United States

**Keywords:** gastric cancer, ERas, autophagy, apoptosis, resistance

## Abstract

Gastric cancer (GC), a common type of malignant cancer, remains the fifth most frequently diagnosed cancer and the third leading cause of cancer-related deaths worldwide. Despite developments in the treatment of GC, the prognosis remains poor. Embryonic stem cell-expressed Ras (ERas), a novel member of the Ras protein family, has recently been identified as an oncogene involved in the tumorigenic growth of embryonic stem cells. A recent study reported that ERas is expressed in most GC cell lines and GC specimens, and it promotes tumorigenicity in GC through induction of the epithelial mesenchymal transition (EMT) and activation of the PI3K/AKT pathway. Here, we found that ERas blocked autophagy flux in BGC-823 and AGS GC cells, which may occur through activation of the AKT/mTOR signaling pathway. Moreover, ERas overexpression suppressed cisplatin-induced apoptosis, and rapamycin treatment significantly attenuated ERas-mediated cisplatin resistance in GC cells. These data suggest that ERas may be a potential therapeutic target to improve the outcomes of GC patients by regulating the autophagy process.

## Introduction

Gastric cancer (GC) is one of the most common types of malignant cancer; it remains the fifth most frequently diagnosed cancer (1033701 new cases in 2018) and the third leading cause of cancer-related deaths (8.2% in total) worldwide (Bray et al., 2018). The incidence of GC is dramatically elevated in eastern Asia (e.g., in the Republic of Korea, which is the country with the highest rates worldwide in both men and women) (Bray et al., 2018). *Helicobacter pylori* is the main risk factor for stomach cancer, and dietary components (foods preserved by salting and low fruit intake), alcohol consumption and active tobacco use are also established risk factors (IARC Working Group on the Evaluation of Carcinogenic Risks to Humans, 2012). Surgery combined with chemotherapy is the primary treatment for GC. However, due to its high chemoresistance, traditional chemotherapeutic drugs, such as cisplatin and fluorouracil only have 10–20% efficacy in treating GC (Kamiyama et al., 2019). The mechanism of high chemoresistance in GC is complex and multifactorial. It is important to explore the molecular mechanism underlying the chemoresistance of GC.

Autophagy is a highly regulated catabolic pathway responsible for the degradation and recycling of cellular components upon various cellular stresses (Cao et al., 2019). Autophagy is a highly conserved process that involves the formation of double membrane vesicles called autophagosomes that engulf cellular proteins and organelles for delivery to the lysosome for degradation (Levy et al., 2017). Autophagy is executed by ATG proteins (ULK1, BECN1, ATG3/5/7/12, and LC3 etc.) and is regulated by several signaling pathways, including PI3K/Akt/mTOR, AMPK, and P53 pathway (Cao et al., 2019). Recently, various studies have shown that autophagy plays a dual role in GC. On one hand autophagy stimulation can act as a protective mechanism to prevent cancer initiation and cell growth (Liang and Jung, 2010). On the other hand, autophagy can also protect some tumor cells against nutrient deprivation and low-oxygen conditions and play an oncogenic function that promotes tumor progression (Katheder et al., 2017).

Embryonic stem cell-expressed Ras (ERas) is a novel member of the Ras protein family that is expressed by the Ras-like gene, which was first found in mouse embryonic stem (ES) cells (Takahashi et al., 2003). ERas was reported to be an oncogene that maintains tumor-like properties in ES cells (Kubota et al., 2010). Normally, ERas is not expressed in somatic cells because of epigenetic transcriptional silencing, but many studies have found that ERas expression exists in GC tissue and several cancer cell lines, including breast cancer, colorectal cancer, and pancreatic cancer (Yashiro et al., 2009). Activating ERas might be associated with tumorigenic growth of somatic cells and might be the molecule responsible for maintaining stem cell-like characteristics in GC (Yasuda et al., 2007). ERas was also shown to promote the activation of the PI3K/AKT pathway and the epithelial mesenchymal transition (EMT) in GC (Aoyama et al., 2010). Furthermore, ERas enhances resistance to CPT-11 in GC via activation of the PI3K/mTOR pathway and NF-κB (Kubota et al., 2011). Since the mTOR signaling pathway is a well-known negative regulator of autophagy, we hypothesized that ERas may also regulate autophagy in GC cells.

In this study, we investigated the role of ERas in regulating autophagy in GC cells. We reported here that ERas overexpression blocked autophagic flux through the AKT/mTOR signaling pathway. Moreover, ERas reduced cisplatin-induced apoptosis in gastric cells and activating autophagy blocks ERas-related resistance to cisplatin. Our findings suggested that ERas might enhance resistance to apoptosis through autophagy suppression in GC cells.

## Materials and Methods

### Cell Culture

The BGC-823 and AGS human GC cell lines, were purchased from Shanghai Cell Biology, Chinese Academy of Sciences (Shanghai, China). BGC-823 and AGS cells were cultured in DMEM supplemented with 10% fetal bovine serum (FBS; HyClone) and penicillin/streptomycin (100 U/ml). All cells were cultured at 37°C in a humidified incubator containing 5% CO_2_.

### Transient Transfection

For the knockdown experiment, plasmids encoding a short hairpin RNAs (shRNAs) targeting ERas and an empty plasmid vector (pSuper) were constructed. We constructed two shRNAs and these interference vectors were named shERas-1 and shERas-2. The sequences of shERas-1 and shERas-2 were 5′-GATCCCCCCATCCAGGATTCCTACTGGTTCAAGAGACCA GTAGGAATCCTGGATGGTTTTTTA-3′ and 5′-GATCCCC GGATTCCTACTGGAAGGAGTTTTCAAGAGAAACTCCTTC CAGTAGGAATCCTTTTTA-3′, respectively, and shRNAs were synthesized by Tsingke, Co., Ltd. (Guangzhou, China). Cells were seeded in 6-well plates and grown to a density of 80% for transfection. The empty plasmid vector and shRNA plasmids were incubated with PEI for 30 min at room temperature. After changing to fresh medium without antibiotics, cells were transfected with 2 μg plasmid per well and incubated overnight. After that, the stable knockdown cell lines were selected with 2 μg/ml puromycin.

### Lentiviral Vector Construction, Lentivirus Packaging and Infection

The ERas amplified from the cDNA of AGS cells was cloned with BamH1 and Not1 restriction sites into a lentiviral pLVX3 vector (a gift from Dr. Changliang Shan, Jinan University), that contained Flag-tag, and was named pLVX3-Flag-ERas.

293T cells were seeded in 10 cm dishes and transfected with the lentiviral expression vector pLVX3-Flag-ERas and the two package vectors psPAX2 and pMD2.G. When the density reached 70 ∼ 80%, culture supernatants (lentivirus) were collected every 24 h for 2 days, filtered through a 0.45 μm pore-size filter and stored at −80°C until use.

Cells were seeded in 6-well plates and grown to a density of 80% for infection. One milliliter of collected culture supernatants (lentivirus) with 10 μg/ml polybrene was added to the wells for 24 h and replaced with fresh medium to culture for another 24 h. Cells were cultured by using fresh medium containing 2 μg/ml puromycin to select the stable cell line for 7 days.

### Real-Time Quantitative PCR

RNA was extracted from cells using RNAiso Plus (Takara Biotechnology, 9109) according to the manufacturer’s instructions. The ABScript II cDNA First Strand Synthesis Kit (ABclonal, RK20400) were used to reverse transcribed the total RNA into cDNA. Real-time PCR was performed using the CFX96 real-time system (Biorad) and SYBR Green select master mix (RK21203, ABclonal). The Actb gene was used as the internal control. Relative gene expression levels were calculated using the 2^–ΔΔ*CT*^ method. The specific primer sequences used in the study are provided in Supplementary Table S1.

### Western Blotting Analysis

Total proteins were extracted from cell cultures using the ice-cold RIPA lysis buffer (Beyotime, P0013B) with proteinase inhibitors. The protein concentrations were determined with a BCA protein kit (Thermo, Cat No. 23225). Samples containing equal amounts of protein were separated by SDS-PAGE, electrotransfer onto a PVDF membrane (Millipore, IPVH00010) and probed with primary antibodies (Supplementary Table S2) at 4°C overnight. The membrane was incubated with secondary antibody at room temperature for 1 h. Protein bands detected by the antibodies were visualized by enhanced chemiluminescence (Beyotime, P0018) and evaluated using Quantity One 1-D Analysis Software (Bio-Rad, Hercules, CA, United States).

### Confocal Fluorescence Microscopy

For autophagy analysis, cells were transfected with GFP-LC3 or mCherry-GFP-LC3 plasmid for 48 h. Cells were fixed in 4% formaldehyde for 15 min subsequently stained with DAPI for 5 min at room temperature and images were captured by a Leica laser scanning confocal microscope (Leica, TCS SP8).

### Cell Apoptosis Assay by Flow Cytometry (FCM)

Embryonic stem cell-expressed Ras overexpressing and normal gastric cells were treated with cisplatin (100 ng/ml for BGC-823 and 50 μg/ml for AGS) or cisplatin plus rapamycin (1 μM) for 12 h before harvesting. Cells were stained with Annexin V and PI (Beyotime, Haimen, Jiangsu, China) and assayed for apoptosis by flow cytometry. In brief, cells were digested with trypsin and washed in PBS. The cells were resuspended with 200 μl Annexin-V binding buffer. Five microliters of Annexin V and 10 μl PI were added and incubated with cells for 10 min in the dark. Cell apoptosis was analyzed by using a FACS Calibur flow cytometer (BD Biosciences, FACSVerse).

### Statistical Analysis

All experiments were repeated at least three times. The data are expressed as the means ± SD. The differences between groups were analyzed by one-way ANOVA using SPSS 19.0 (SPSS Inc., Chicago, IL, United States). *p*-values of less than 0.05 were regarded as statistically significant.

## Results

### ERas Blocks Autophagic Flux in Gastric Cancer Cells

**FIGURE 1:**
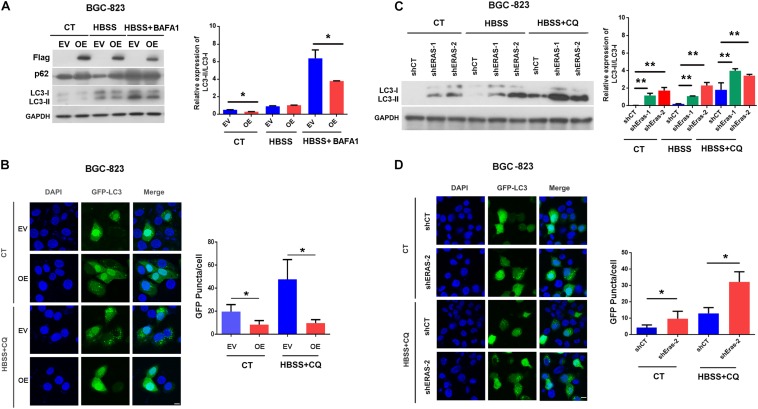
Embryonic stem cell-expressed Ras (ERas) blocks autophagy in gastric cancer cells. **(A)** Representative western blots of Flag-ERas, p62, LC3B in ERas stable overexpressed and control BGC-823 cells, quantification on right panel (EV, empty vector; OE, ERas overexpression, Data represent as mean ± SD of three individual experiments, ^∗^*p* < 0.05). **(B)** Representative images and quantitative densitometric results of GFP-LC3 puncta in ERas stable overexpressed or control BGC-823 cells upon HBSS (Hank’s Balanced Salt Solution) or HBSS plus CQ treatment (chloroquine, 50 μM for 12 h, Scale bar = 10 μm; Data represent as mean ± SD of three individual experiments, ^∗^*p* < 0.05). **(C)** Representative western blots of LC3B in ERas knockdown and control BGC-823 cells, quantification on right panel (ERas knockdown: shERas-1 and shERas-2, Data represent as mean ± SD of three individual experiments, ^∗∗^*p* < 0.01). **(D)** Representative images and quantitative densitometric results of GFP-LC3 puncta in ERas knockdown or control BGC-823 cells upon HBSS or HBSS plus CQ treatment (chloroquine, 50 μM for 12 h, Scale bar = 10 μm; Data represent as mean ± SD of three individual experiments, ^∗^*p* < 0.05).

To investigate the potential roles of ERas in autophagy, we performed gain-of-function and loss-of-function experiments to examine the role of ERas in regulating autophagy in gastric cells. We generated a stably overexpressed ERas gastric cell line and ERas knockdown cells (Supplementary Figure S1). Overexpression of ERas significantly decreased the lipidation of LC3B (the ratio of LC3-II/LC3-I), but increased the autophagy substrate p62 under normal and starvation conditions in BGC-823 and AGS cells (Figure 1A and Supplementary Figure S2A). Moreover, ERas overexpression dramatically decreased autophagosome formation (GFP-LC3 puncta) in both BGC-823 and AGS cells compared with that of the control cells (Figures 1B and Supplementary Figure S2B). However, knockdown of ERas significantly promoted lipidation of LC3B and autophagosome formation (GFP-LC3 puncta) in BGC-823 and AGS cells (Figures 1C,D and Supplementary Figures S2C,D). However, overexpression of ERas had no effect on the transcription levels of autophagy-related genes (Supplementary Figure S3). Furthermore, we found that ERas overexpression also decreased lipidation of LC3B and autophagosome formation upon BafA1 [a inhibitor of vacuolar type H+-ATPase (V-ATPase) that prevent the acidification of lysosomes] or chloroquine (CQ, an inhibitor of autophagosome-lysosome fusion) treatment (Figures 1A,B). These data suggest that ERas may block autophagy.

**FIGURE 2:**
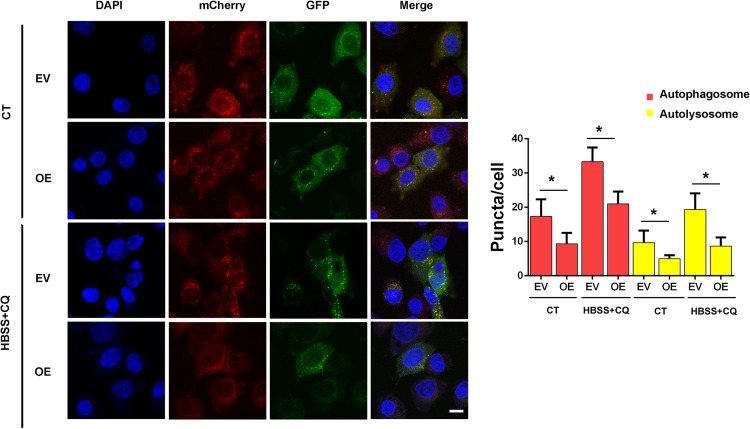
ERas blocks autophagic flux in BGC-823 cells. Detection and quantification of the autophagic flux with the mCherry-GFP-LC3 reporter in ERas stable overexpressed and control BGC-823 cells upon control or HBSS plus CQ treatment. Cells were treated with HBSS plus CQ (chloroquine, 50 μM for 12 h). autophagosomes (GFP^+^/mCherry^+^, yellow puncta), autolysosomes (GFP^–^/mCherry^+^, red puncta). (EV, empty vector; OE, ERas overexpression; Scale bar = 10 μm; ^∗^ *p* < 0.05).

Next, we examined whether ERas regulates autophagic flux by using the mCherry-GFP-LC3 reporter plasmid. Overexpression of ERas not only decreased autolysosomes (mCherry^+^/GFP^–^, red puncta) under both the normal and starvation conditions, but also decreased the accumulation of autophagosomes (mCherry^+^/GFP^+^, yellow puncta) under CQ treatment in BGC-823 cells (Figure 2). Together, these results demonstrated that ERas blocks autophagic flux in both normal and stress conditions.

### ERas Activates the AKT/mTOR Pathway in Gastric Cancer Cells

**FIGURE 3:**
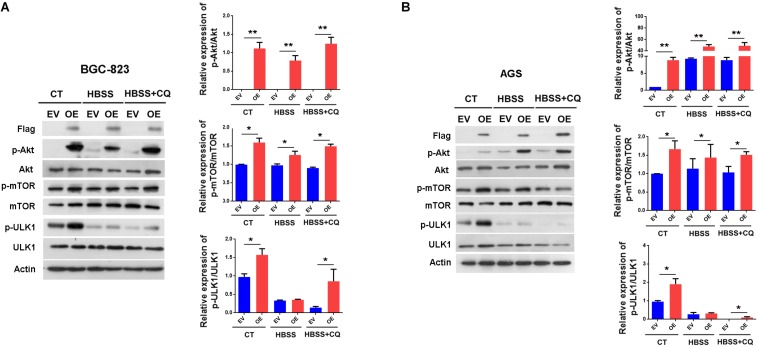
ERas activates AKT/mTOR pathway in gastric cancer cells. **(A,B)** Representative western blots of p-Akt (Ser-473), Akt, p-mTOR (Ser-2448), mTOR, p-ULK1 (Ser-757), ULK1 in ERas stable overexpressed and control BGC-823 **(A)** or AGS **(B)** cells, quantification on right panel. Cells were treated with HBSS or HBSS plus CQ (50 μM) for 12 h. Data represent as mean ± SD of three individual experiments, ^∗^*p* < 0.05, ^∗∗^*p* < 0.01, compared with the control.

To explore the signaling pathway responsible for the suppression of autophagy by ERas, we examined the involvement of the mTOR signaling pathway, which is a well-known negative regulator of autophagy. As shown in Figure 3, ERas overexpression significantly increased phosphorylation levels of both mTOR (Ser2448) and its substrate (ULK1-Ser757) under both normal and starvation conditions in BGC-823 and AGS cells, compared to those of control cells. Moreover, ERas overexpression increased the phosphorylation of AKT-Ser473 (Figure 3). This is consistent with a previous study showing that ERas interacted with PI3K and activated its downstream signaling (Kubota et al., 2011). Akt is an upstream positive regulator of the mTOR via direct phosphorylation and inhibition of TSC2, which is a negative regulator of mTOR, as well as through regulating cellular ATP level and AMPK activity (Hahn-Windgassen et al., 2005). These data suggested that ERas activates the AKT/mTOR pathway, which may contribute to the ERas-mediated autophagy inhibition in GC cell.

### ERas Blocks Cisplatin-Induced Apoptosis in Gastric Cancer Cells

**FIGURE 4:**
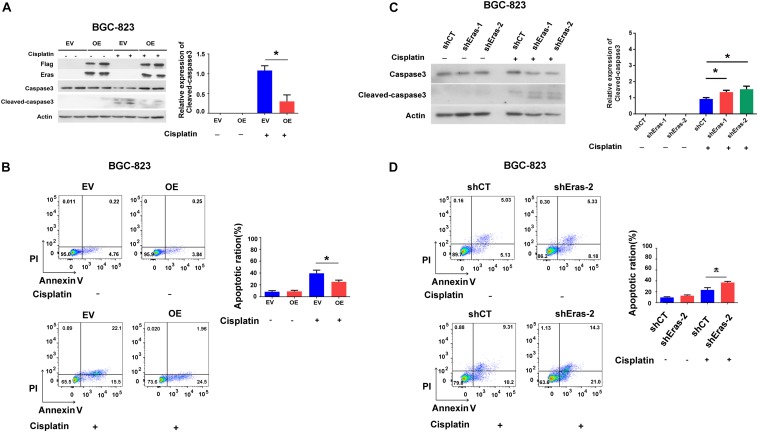
ERas blocks cisplatin-induced apoptosis in gastric cells. **(A)** Representative western blots of full length caspase-3 and cleaved-caspase 3 in ERas stable overexpressed and control BGC-823 cells, quantification of cleaved-caspase 3 on right panel (cisplatin 100 ng/ml for 12 h, Data represent as mean ± SD of three individual experiments, ^∗^*p* < 0.05). **(B)** Cell apoptotic ratio of ERas stable overexpressed and control BGC-823 cell was determined by flow cytometry (FACS) with Annexin V – FITC and PI double staining, quantification of apoptotic ratio on right panel (cisplatin 100 ng/ml for 12 h, ^∗^*p* < 0.05). **(C)** Representative western blots of full length caspase3 and cleaved-caspase 3 in ERas knockdown and control BGC-823 cells, quantification of cleaved-caspase 3 on right panel (cisplatin 100 ng/ml for 12 h, Data represent as mean ± SD of three individual experiments, ^∗^*p* < 0.05 compared with shCT group). **(D)** Cell apoptotic ratio of ERas knockdown and control BGC-823 cell was determined by flow cytometry (FACS) with Annexin V – FITC and PI double staining, quantification of apoptotic ratio on right panel (cisplatin 100 ng/ml for 12 h, ^∗^*p* < 0.05).

Subsequently, we asked if ERas regulates apoptosis in GC cells, and we found that overexpression of ERas dramatically repressed cisplatin-induced caspase-3 cleavage in both BGC-823 and AGS cell lines compared with that of control cells (Figure 4A and Supplementary Figure S4A). Consistently, the flow cytometry results also showed that overexpression of ERas significantly repressed apoptosis (Annexin V positive cells) in BGC-823 (from 35 to 26%) and AGS (from 62 to 34%) cells (Figure 4B and Supplementary Figure S4B). However, ERas knockdown significantly promoted cisplatin-induced caspase-3 cleavage as well as apoptosis in both BGC-823 and AGS cells (Figures 4C,D and Supplementary Figures S4C,D). These data suggest that ERas repressed cisplatin-induced apoptosis in GC cell lines.

### ERas Blocks Cisplatin-Induced Apoptosis by Suppressing Autophagy

**FIGURE 5:**
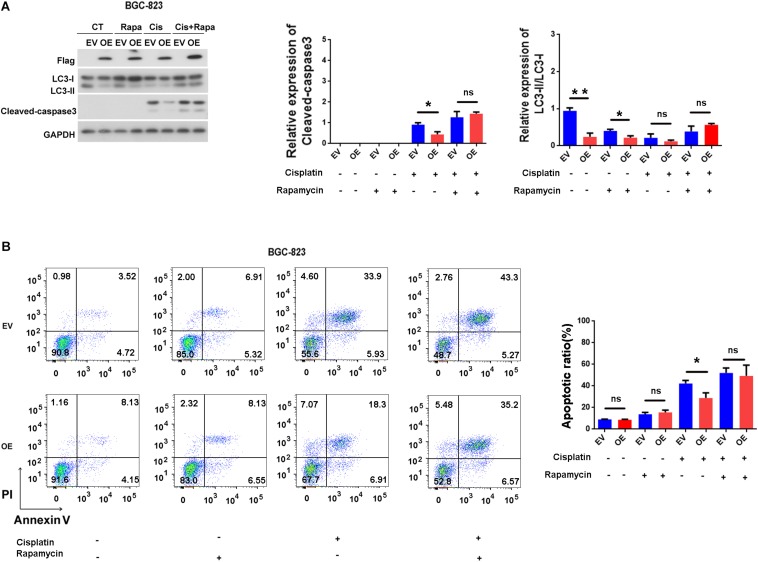
ERas blocks cisplatin-induced apoptosis through suppressing autophagy in BGC-823 cells. **(A)** Representative western blots of LC3B and cleaved-caspase 3 in ERas stable overexpressed and control BGC-823 cells upon cisplatin or rapamycin treatments, quantification of cleaved-caspase 3 and LC3B on right panel (cisplatin 100 ng/ml, rapamycin 1 μM for 12 h, Data represent as mean ± SD of three individual experiments, ^∗^*p* < 0.05, ^∗∗^*p* < 0.01). **(B)** Cell apoptotic ratio of ERas stable overexpressed and control BGC-823 cell was determined by flow cytometry (FACS) with Annexin V – FITC and PI double staining, quantification on right panel (cisplatin 100 ng/ml, rapamycin 1 μM for 12 h, Data represent as mean ± SD of three individual experiments, ^∗^*p* < 0.05).

There is complex connection between apoptosis and autophagy (Gump and Thorburn, 2011). Thus, we examined whether ERas blocks apoptosis through autophagy. As shown in Figure 5A, Rapamycin only treatment have no effect on caspase-3 cleavage and apoptosis (Figures 5A,B), but ERas overexpressing cells exhibited decreased cisplatin-induced caspase-3 cleavage compared with control cells (Figure 5A). However, when treated with cisplatin and rapamycin together, the protein levels of cleaved-caspase-3 were increased in both control and ERas-overexpressing cells compared with those of the control group. Further addition of rapamycin promoted cisplatin-induced caspase-3 cleavage in ERas-overexpressing cells similar to that of control BGC-823 cells (Figure 5A). Surprisingly, cisplatin treatment decreased lipidation of LC3B, and blocked ERas-mediated autophagy suppression (Figure 5A). Furthermore, flow cytometry analysis results showed that the number of apoptotic cells significantly decreased in ERas-overexpressing cells upon cisplatin treatment compared to that of the control group. However, there were no differences between ERas-overexpressing and control BGC cells with cisplatin and rapamycin cotreatment (Figure 5B). These experimental data suggest that activation of autophagy by rapamycin can increase cisplatin-induced apoptosis in ERas-overexpressing BGC-823 cells.

## Discussion

Gastric cancer is one of the most common cancers worldwide, especially in Asia, with only a 5 year survival rate of diagnosis (Liu et al., 2019; Rugge et al., 2019). Despite developments in the treatment of GC, the prognosis remains poor. Therefore, there is a need to develop novel and effective therapeutic targets to improve the outcomes of patients with GC.

Embryonic stem cell-expressed Ras is a recently identified oncogene involved in the tumorigenic growth of embryonic stem cells (Takahashi et al., 2003). ERas mRNA is expressed in several cancer cell lines, including colorectal carcinomas, pancreatic carcinomas, and breast carcinomas, but not in normal cell lines (Yasuda et al., 2007; Suarez-Cabrera et al., 2018). In addition, ERas mRNA is expressed in most GC cell lines to different degrees, and ERas was also detected in most clinical GC specimens, but not in adjacent tissues (Kubota et al., 2010). Immunohistochemistry also showed that ERas is expressed in 38.7% (55/142) of human GC tissues but not in adjacent tissues (Takahashi et al., 2003). ERas upregulates transcription regulatory factors, including ZFHX1A, ZFHX1B, and TCF3, which repress E-cadherin expression in GC (Kubota et al., 2010). A recent study reported that ERas promotes tumorigenicity of GC through activation of the PI3K/AKT pathway and is associated with GC metastasis (Aoyama et al., 2010). Raf/MAPK is another best characterized Ras effector, which regulates cell proliferation, differentiation and cell death (Molina and Adjei, 2006). However, consistent with previous studies showing that ERas cannot activate MAPK/ERK pathway (Nakhaei-Rad et al., 2016; Suarez-Cabrera et al., 2018), we also found that ERas overexpression have no effect on the phosphorylation of p38 and JNK (Supplementary Figure S5). These results suggested that ERas activates PI3K/AKT but not MAPK signaling pathway in GC cells. Our results demonstrated that ERas blocks autophagy flux in both BGC-823 and AGS cells. Moreover, ERas activates the AKT/mTOR pathway, which may contribute to the ERas-mediated autophagy inhibition in GC cells (Figures 1–3).

Aberrant or excessive autophagic activity may produce cytotoxicity, resulting in autophagic cell death and suppressing tumor development in GC cells (Qian and Yang, 2016). Fluorouracil (5-FU) may induce cell proliferation arrest and autophagic cell death in GC cells by suppressing miR-30, resulting in upregulating Beclin1 (Yang and Pan, 2015). On the other hand, autophagy promotes tumor progression and prevents cancer cells from undergoing drug-induced apoptosis. Oxaliplatin-induced autophagy can partially antagonize apoptotic cell death in GC cells (Xu et al., 2011). In addition, autophagy facilitates Aquaporin 3 -mediated chemoresistance to cisplatin in GC cells, whereas inhibition of autophagy by chloroquine significantly enhances cisplatin chemosensitivity in GC cells (Dong et al., 2016). In addition to apoptosis, cisplatin has been reported to induce autophagy in lung, esophageal, and ovarian cancer cells (Xu et al., 2012; Yu et al., 2014; Wu et al., 2015). Moreover, low dose of cisplatin also could induce autophagy in lung-cancer cells (5 μM) and bladder cancer cells (6.25 μM) (Cho et al., 2014; Lin et al., 2017). However, inhibitor of autophagy promotes cisplatin-induced apoptosis (Xu et al., 2012; Chen et al., 2018). In our study, we found that ERas blocked cisplatin-induced apoptosis in GC cells (BGC-823 and AGS), rapamycin treatment significantly attenuated ERas-mediated cisplatin resistance in GC cells (Figures 4, 5). Surprisingly, cisplatin treatment decreased lipidation of LC3B, and blocked ERas-mediated autophagy suppression (Figure 5A). This suggest that very low concentration of cisplatin (100 ng/ml = 0.33 μM) may block autophagy instead of inducing autophagy in GC cells.

Autophagy and apoptosis are both physiological processes essential for organismal homeostasis. The relationship between autophagy and apoptosis is complex, and many stimuli can induce both processes (Su et al., 2013). In most cases, autophagy functions as a survival mechanism through which the cells adapt to such conditions, escaping cell death induced by apoptosis. However, in other situations, both autophagy and apoptosis can promote cell death (Nikoletopoulou et al., 2013). In addition, autophagy can also promote apoptosis, and autophagy promotes apoptosis of mesenchymal stem cells under an inflammatory microenvironment (Dang et al., 2015). Oleifolioside B-mediated autophagy functions as a death mechanism by promoting apoptosis and autophagic cell death in A549 cells (Jin et al., 2013). Luteolin induces apoptosis in human liver cancer SMMC-7721 cells, partially via autophagy (Cao et al., 2017).

In conclusion, our results demonstrate that ERas expression enhanced resistance to cisplatin-induced apoptosis and that ERas suppressed autophagic flux through the Akt/mTOR pathway. Moreover, we found that rapamycin treatment significantly attenuated ERas-mediated cisplatin resistance in GC cells. These data suggest that ERas may be a potential therapeutic target to improve the outcomes of GC patients by regulating the autophagy process.

## Data Availability Statement

The raw data supporting the conclusion of this article will be made available by the authors, without undue reservation, to any qualified researcher.

## Author Contributions

QZ and JL supervised the project and designed the experiments. HT, WW, XM, JT, MW, and WJ performed the experiments. HT, WW, JL, and PL analyzed the experiments and provided technical support and discussions. HT, JL, and QZ wrote the manuscript. All the authors reviewed the manuscript.

## Conflict of Interest

The authors declare that the research was conducted in the absence of any commercial or financial relationships that could be construed as a potential conflict of interest.

## Supplementary Material

FIGURE S1 ShRNA mediates ERas knockdown in BGC-823 and AGS cells. **(A)** mRNA expression of ERas in BGC-823 and AGS cells (ERas knockdown: shERas-1 and shERas-2, Data represent as mean ± SD of three individual experiments, ^∗^*p* < 0.05, ^∗∗^*p* < 0.01). **(B)** Representative western blots of ERas expression after shRNA transfection 48 h in BGC-823 cells.

FIGURE S2 ERas blocks autophagy in AGS cells. **(A)** Representative western blots of Flag-ERas, p62, LC3B in ERas stable overexpressed and control AGS cells, quantification on right panel (EV: empty vector, OE: ERas overexpression, Data represent as mean ± SD of three individual experiments, ^∗^*p* < 0.05). **(B)** Representative images and quantitative densitometric results of GFP-LC3 puncta in control, ERas stable overexpressed AGS cells upon HBSS or HBSS plus CQ treatment (chloroquine, 50 μM for 12 h, Scale bar =10 μm; Data represent as mean ± SD of three individual experiments, ^∗^*p* < 0.05). **(C)** Representative western blots of, LC3B in ERas knockdown and control BGC-823 cells, quantification on right panel (ERas knockdown: shERas-1 and shERas-2, Data represent as mean ± SD of three individual experiments, ^∗^*p* < 0.05, ^∗∗^*p* < 0.01). **(D)** Representative images and quantitative densitometric results of GFP-LC3 puncta in control or ERas knockdown AGS cells upon HBSS or HBSS plus CQ treatment (chloroquine, 50 μM for 12 h, Scale bar = 10 μm; Data represent as mean ± SD of three individual experiments, ^∗^*p* < 0.05).

FIGURE S3 mRNA expression of autophagy related genes in ERas stable overexpressed (OE) or control (EV) BGC-823 cells. (Data represent as mean ± SD of three individual experiments, ^∗∗∗^*p* < 0.001, compared with the control).

FIGURE S4 ERas blocks cisplatin-induced apoptosis in AGS cells. **(A)** Representative western blots of full length caspase3 and cleaved-caspase 3 in ERas stable overexpressed and control AGS cells, quantification of cleaved-caspase 3 on right panel (cisplatin 50 μg/ml for 12 h, Data represent as mean ± SD of three individual experiments, ^∗^*p* < 0.05). **(B)** Cell apoptotic ratio of ERas stable overexpressed and control AGS cells were determined by flow cytometry (FACS) with Annexin V-FITC and PI double staining, quantification of apoptotic ratio on right panel (cisplatin 50 μg/ml for 12 h, ^∗^*p* < 0.05). **(C)** Representative western blots of full length caspase3 and cleaved-caspase 3 in ERas knockdown and control AGS cells, quantification of cleaved-caspase 3 on right panel (cisplatin 50 μg/ml for 12 h, Data represent as mean ± SD of three individual experiments, ^∗^*p* < 0.05). **(D)** Cell apoptotic ratio of ERas knockdown and control AGS cells were determined by flow cytometry (FACS) with Annexin V-FITC and PI double staining, quantification of apoptotic ratio on right panel (cisplatin 50 μg/ml for 12 h, ^∗^*p* < 0.05).

FIGURE S5 ERas does not activate MAPK signaling pathway in BGC-823 cells. Representative western blots of p-p38 and p-JNK in ERas stable overexpressed and control BGC-823 cells, quantification of p-p38 and p-JNK on right panel (Data represent as mean ± SD of three individual experiments, ns = not significant).

TABLE S1 Sequences of primers used in the present study.

TABLE S2 Primary antibodies used in the present study.

DATA SHEET S1 Raw data.

## References

[B1] Aoyama M. Kataoka H. Kubota E. Tada T. Asai K. (2010). Resistance to chemotherapeutic agents and promotion of transforming activity mediated by embryonic stem cell-expressed Ras (ERas) signal in neuroblastoma cells. *Int. J. Oncol.* 37 1011–1016. 20811723 10.3892/ijo_00000752

[B2] Bray F. Ferlay J. Soerjomataram I. Siegel R. L. Torre L. A. Jemal A. (2018). Global cancer statistics 2018: GLOBOCAN estimates of incidence and mortality worldwide for 36 cancers in 185 countries. *CA Cancer J. Clin.* 68 394–424. 10.3322/caac.21492 30207593

[B3] Cao Y. Luo Y. Zou J. Ouyang J. Cai Z. Zeng X. (2019). Autophagy and its role in gastric cancer. *Clin. Chim. Acta* 489 10–20. 10.1016/j.cca.2018.11.028 30472237

[B4] Cao Z. Zhang H. Cai X. Fang W. Chai D. Wen Y. (2017). Luteolin promotes cell apoptosis by inducing autophagy in hepatocellular carcinoma. *Cell. Physiol. Biochem.* 43 1803–1812. 10.1159/000484066 29049999

[B5] Chen J. Zhang L. Zhou H. Wang W. Luo Y. Yang H. (2018). Inhibition of autophagy promotes cisplatin-induced apoptotic cell death through Atg5 and Beclin 1 in A549 human lung cancer cells. *Mol. Med. Rep.* 17 6859–6865. 10.3892/mmr.2018.8686 29512762

[B6] Cho K. H. Park J. H. Kwon K. B. Lee Y. R. So H. S. Lee K. K. (2014). Autophagy induction by low-dose cisplatin: the role of p53 in autophagy. *Oncol. Rep.* 31 248–254. 10.3892/or.2013.2809 24173208

[B7] Dang S. Yu Z. M. Zhang C. Y. Zheng J. Li K. L. Wu Y. (2015). Autophagy promotes apoptosis of mesenchymal stem cells under inflammatory microenvironment. *Stem Cell Res. Ther.* 6:247. 10.1186/s13287-015-0245-4 26670667 PMC4681177

[B8] Dong X. Wang Y. Zhou Y. Wen J. Wang S. Shen L. (2016). Aquaporin 3 facilitates chemoresistance in gastric cancer cells to cisplatin via autophagy. *Cell Death Discov.* 2:16087. 27867537 10.1038/cddiscovery.2016.87 PMC5107998

[B9] Gump J. M. Thorburn A. (2011). Autophagy and apoptosis: what is the connection? *Trends Cell Biol.* 21 387–392. 10.1016/j.tcb.2011.03.007 21561772 PMC3539742

[B10] Hahn-Windgassen A. Nogueira V. Chen C. C. Skeen J. E. Sonenberg N. Hay N. (2005). Akt activates the mammalian target of rapamycin by regulating cellular ATP level and AMPK activity. *J. Biol. Chem.* 280 32081–32089. 10.1074/jbc.m502876200 16027121

[B11] IARC Working Group on the Evaluation of Carcinogenic Risks to Humans (2012). Personal habits and indoor combustions. Volume 100 E. A review of human carcinogens. *IARC Monogr. Eval. Carcinog. Risks Hum.* 100 1–538. PMC4781577 23193840

[B12] Jin C. Y. Yu H. Y. Park C. Han M. H. Hong S. H. Kim K. S. (2013). Oleifolioside B-mediated autophagy promotes apoptosis in A549 human non-small cell lung cancer cells. *Int. J. Oncol.* 43 1943–1950. 10.3892/ijo.2013.2143 24141596

[B13] Kamiyama K. Sawada S. Fukuda T. Yamaguchi T. Kato N. Kojima A. (2019). A case of long-term survival following two-stage surgery for perforated advanced gastric cancer caused by chemotherapy. *Gan To Kagaku Ryoho* 46 701–704. 31164510

[B14] Katheder N. S. Khezri R. Schultz S. W. Jain A. Rahman M. M. Schink K. O. (2017). Microenvironmental autophagy promotes tumour growth. *Nature* 541 417–420. 10.1038/nature20815 28077876 PMC5612666

[B15] Kubota E. Kataoka H. Aoyama M. Mizoshita T. Mori Y. Shimura T. (2010). Role of ES cell-expressed Ras (ERas) in tumorigenicity of gastric cancer. *Am. J. Pathol.* 177 955–963. 10.2353/ajpath.2010.091056 20566745 PMC2913346

[B16] Kubota E. Kataoka H. Tanaka M. Okamoto Y. Ebi M. Hirata Y. (2011). ERas enhances resistance to CPT-11 in gastric cancer. *Anticancer Res.* 31 3353–3360. 21965746

[B17] Levy J. M. M. Towers C. G. Thorburn A. (2017). Targeting autophagy in cancer. *Nat. Rev. Cancer* 17 528–542. 28751651 10.1038/nrc.2017.53 PMC5975367

[B18] Liang C. Jung J. U. (2010). Autophagy genes as tumor suppressors. *Curr. Opin. Cell Biol.* 22 226–233. 10.1016/j.ceb.2009.11.003 19945837 PMC2854193

[B19] Lin J. F. Lin Y. C. Tsai T. F. Chen H. E. Chou K. Y. Hwang T. I. (2017). Cisplatin induces protective autophagy through activation of BECN1 in human bladder cancer cells. *Drug Design Dev. Ther.* 11 1517–1533. 10.2147/DDDT.S126464 28553083 PMC5439993

[B20] Liu R. Sobue T. Kitamura T. Kitamura Y. Ishihara J. Kotemori A. (2019). Dietary acrylamide intake and risk of esophageal, gastric, and colorectal cancer: the Japan public health center-based prospective study. *Cancer Epidemiol. Biomark. Prev.* 28 1461–1468. 10.1158/1055-9965.EPI-18-1259 31186264

[B21] Molina J. R. Adjei A. A. (2006). The Ras/Raf/MAPK pathway. *J. Thorac. Oncol.* 1 7–9. 10.1016/s1556-0864(15)31506-9 17409820

[B22] Nakhaei-Rad S. Nakhaeizadeh H. Gotze S. Kordes C. Sawitza I. Hoffmann M. J. (2016). The role of embryonic stem cell-expressed ras (ERAS) in the maintenance of quiescent hepatic stellate cells. *J. Biol. Chem.* 291 8399–8413. 10.1074/jbc.M115.700088 26884329 PMC4861415

[B23] Nikoletopoulou V. Markaki M. Palikaras K. Tavernarakis N. (2013). Crosstalk between apoptosis, necrosis and autophagy. *Biochim. Biophys. Acta* 1833 3448–3459. 10.1016/j.bbamcr.2013.06.001 23770045

[B24] Qian H. R. Yang Y. (2016). Functional role of autophagy in gastric cancer. *Oncotarget* 7 17641–17651. 10.18632/oncotarget.7508 26910278 PMC4951239

[B25] Rugge M. Sacchi D. Graham D. Y. Genta R. M. (2019). Secondary prevention of gastric cancer: merging the endoscopic atrophic border with OLGA staging. *Gut* 10.1136/gutjnl-2019-319107 [Epub ahead of print]. 31186295

[B26] Su M. Mei Y. Sinha S. (2013). Role of the crosstalk between autophagy and apoptosis in cancer. *J. Oncol.* 2013:102735. 10.1155/2013/102735 23840208 PMC3687500

[B27] Suarez-Cabrera C. de la Pena B. Gonzalez L. L. Page A. Martinez-Fernandez M. Casanova M. L. (2018). The Ras-related gene ERAS is involved in human and murine breast cancer. *Sci. Rep.* 8:13038. 10.1038/s41598-018-31326-4 30158566 PMC6115423

[B28] Takahashi K. Mitsui K. Yamanaka S. (2003). Role of ERas in promoting tumour-like properties in mouse embryonic stem cells. *Nature* 423 541–545. 10.1038/nature01646 12774123

[B29] Wu H. M. Jiang Z. F. Ding P. S. Shao L. J. Liu R. Y. (2015). Hypoxia-induced autophagy mediates cisplatin resistance in lung cancer cells. *Sci. Rep.* 5:12291. 10.1038/srep12291 26201611 PMC4511870

[B30] Xu L. Qu X. J. Liu Y. P. Xu Y. Y. Liu J. Hou K. Z. (2011). Protective autophagy antagonizes oxaliplatin-induced apoptosis in gastric cancer cells. *Chin. J. Cancer* 30 490–496. 10.5732/cjc.010.10518 21718595 PMC4013424

[B31] Xu Y. Yu H. Qin H. Kang J. Yu C. Zhong J. (2012). Inhibition of autophagy enhances cisplatin cytotoxicity through endoplasmic reticulum stress in human cervical cancer cells. *Cancer Lett.* 314 232–243. 10.1016/j.canlet.2011.09.034 22019047

[B32] Yang C. Pan Y. (2015). Fluorouracil induces autophagy-related gastric carcinoma cell death through Beclin-1 upregulation by miR-30 suppression. *Tumour Biol.* 10.1007/s13277-015-3775-6 [Epub ahead of print]. 26209295

[B33] Yashiro M. Yasuda K. Nishii T. Kaizaki R. Sawada T. Ohira M. (2009). Epigenetic regulation of the embryonic oncogene ERas in gastric cancer cells. *Int. J. Oncol.* 35 997–1003. 19787253 10.3892/ijo_00000414

[B34] Yasuda K. Yashiro M. Sawada T. Ohira M. Hirakawa K. (2007). ERas oncogene expression and epigenetic regulation by histone acetylation in human cancer cells. *Anticancer Res.* 27 4071–4075. 18225573

[B35] Yu L. Gu C. Zhong D. Shi L. Kong Y. Zhou Z. (2014). Induction of autophagy counteracts the anticancer effect of cisplatin in human esophageal cancer cells with acquired drug resistance. *Cancer Lett.* 355 34–45. 10.1016/j.canlet.2014.09.020 25236911

