# Supplementary material for: ERas Enhances Resistance to Cisplatin-Induced Apoptosis by Suppressing Autophagy in Gastric Cancer Cell
Source: Front Cell Dev Biol. 2020 Jan 21;7:375. doi: 10.3389/fcell.2019.00375 (PMC7005724; doi:10.3389/fcell.2019.00375)
Supplement: FIGURE S1 — ShRNA mediates ERas knockdown in BGC-823 and AGS cells. (A) mRNA expression of ERas in BGC-823 and AGS cells (ERas knockdown: shERas-1 and shERas-2, Data represent as mean ± SD of three individual experiments, ∗p < 0.05, ∗∗p < 0.01). (B) Representative western blots of ERas expression after shRNA transfection 48 h in BGC-823 cells. [file Data_Sheet_2.pdf]

Supplimentary figures:

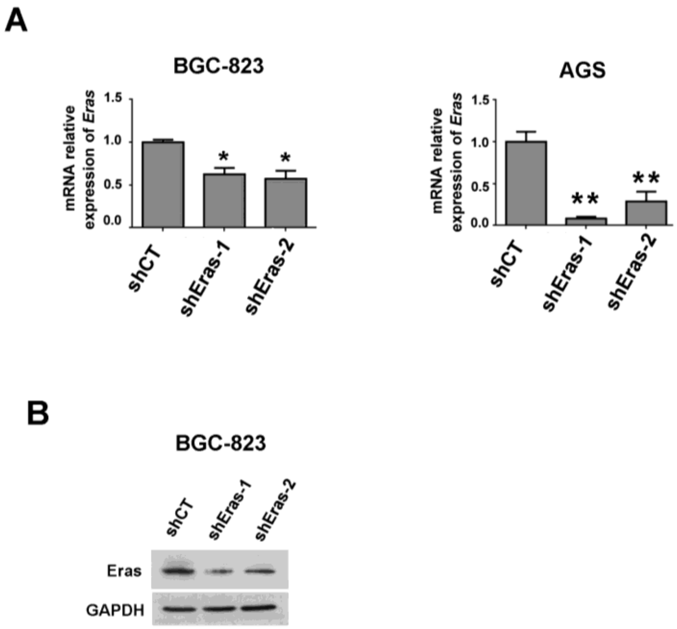

Figure S1. ShRNA mediates ERas knockdown in BGC-823 and AGS cells. A. mRNA expression of ERas in BGC-823 and AGS cells (ERas knockdown: shERas-1 and shERas-2, Data represent as mean ± SD of three individual experiments, *$p < 0.05$, **$p < 0.01$). **B.** Representative western blots of ERas expression after shRNA transfection 48h in BGC-823 cells.

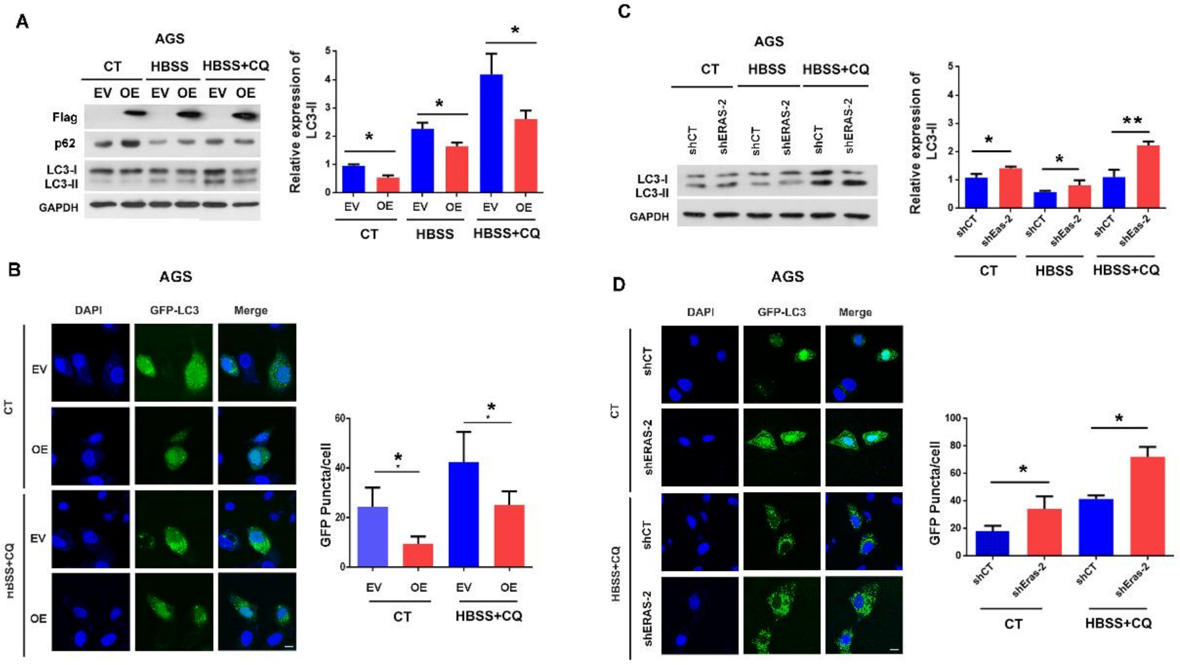

Figure S2. ERas blocks autophagy in AGS cells. A. Representative western blots of Flag-ERas,

p62, LC3B in ERas stable overexpressed and control AGS cells, quantification on right panel (EV: empty vector, OE: ERas overexpression, Data represent as mean ± SD of three individual experiments, *$p < 0.05$). **B.** Representative images and quantitative densitometric results of GFP-LC3 puncta in control, ERas stable overexpressed AGS cells upon HBSS or HBSS plus CQ treatment (chloroquine, 50 μM for 12 h, Scale bar =10 μm; Data represent as mean ± SD of three individual experiments, *$p < 0.05$). **C.** Representative western blots of, LC3B in ERas knockdown and control BGC-823 cells, quantification on right panel (ERas knockdown: shERas-1 and shERas-2, Data represent as mean ± SD of three individual experiments, *$p < 0.05$, **$p < 0.01$). **D.** Representative images and quantitative densitometric results of GFP-LC3 puncta in control or ERas knockdown AGS cells upon HBSS or HBSS plus CQ treatment (chloroquine, 50 μM for 12 h, Scale bar =10 μm; Data represent as mean ± SD of three individual experiments, *$p < 0.05$).

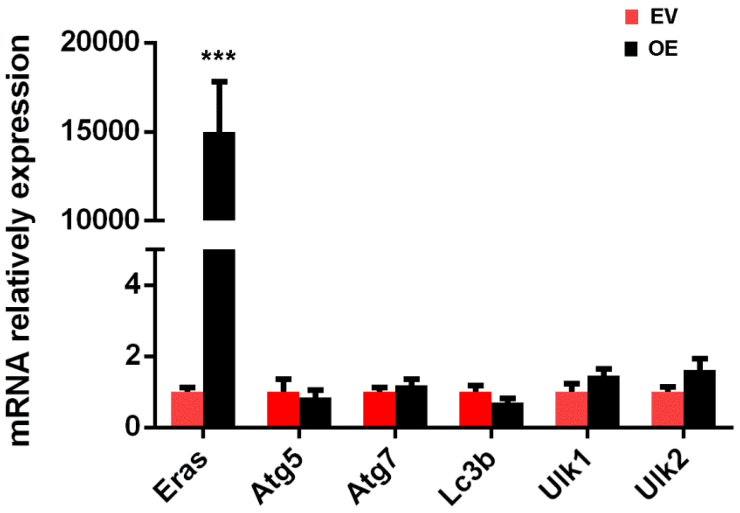

Figure S3. mRNA expression of autophagy related genes in ERas stable overexpressed (OE) or control (EV) BGC-823 cells. (Data represent as mean ± SD of three individual experiments, ***$p < 0.001$, compared with the control).

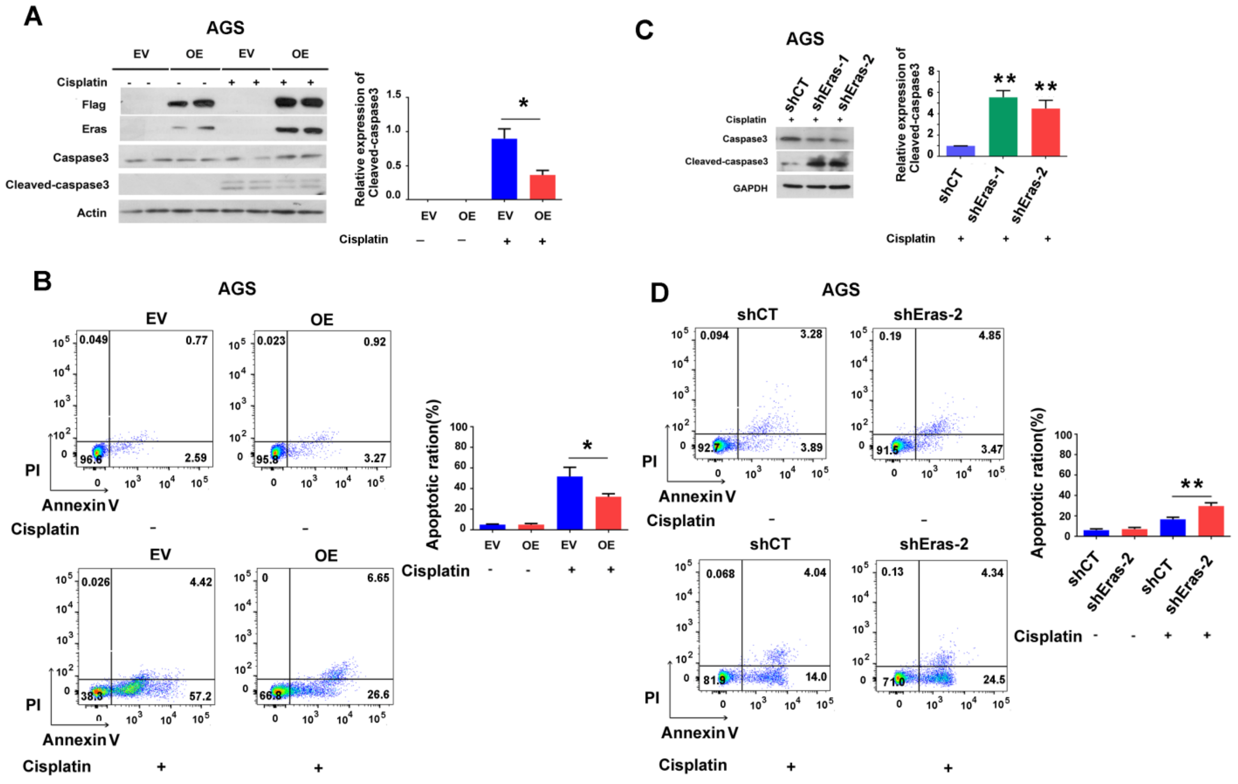

Figure S4. ERas blocks cisplatin-induced apoptosis in AGS cells. A. Representative western blots of full length caspase3 and cleaved-caspase 3 in ERas stable overexpressed and control AGS cells, quantification of cleaved-caspase 3 on right panel (cisplatin 50 μg/ml for 12h, Data represent as mean ± SD of three individual experiments, **p* < 0.05). **B.** Cell apoptotic ratio of ERas stable overexpressed and control AGS cells were determined by flow cytometry (FACS) with Annexin V-FITC and PI double staining, quantification of apoptotic ratio on right panel (cisplatin 50 μg/ml for 12 h, **p* < 0.05). **C.** Representative western blots of full length caspase3 and cleaved-caspase 3 in ERas knockdown and control AGS cells, quantification of cleaved-caspase 3 on right panel (cisplatin 50 μg/ml for 12 h, Data represent as mean ± SD of three individual experiments, **p* < 0.05). **D.** Cell apoptotic ratio of ERas knockdown and control AGS cells were determined by flow cytometry (FACS) with Annexin V-FITC and PI double staining, quantification of apoptotic ratio on right panel (cisplatin 50 μg/ml for 12 h, **p* < 0.05)

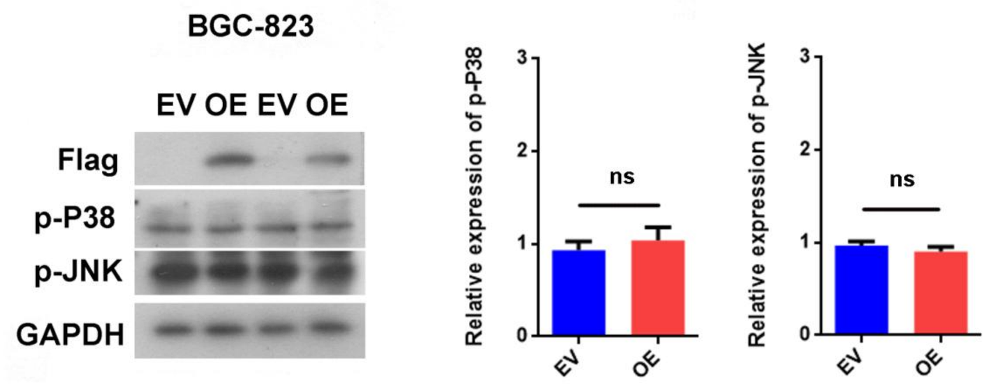

Figure S5. ERas does not activate MAPK signaling pathway in BGC-823 cells. Representative western blots of p-p38 and p-JNK in ERas stable overexpressed and control BGC-823 cells, quantification of p-p38 and p-JNK on right panel (Data represent as mean ± SD of three individual experiments, ns = not significant).

Supplimentary Tables

Table 1. Sequences of primers used in the present study

Gene	Forward	Reverse
ERAS	AGTACAAGGCTGTGGTGGTG	AGTAGGAATCCTGGATGGTGGG
ATG5	GGGTCCCTCTTGGGGTACAT	ACCACACATCTCGAAGCACA
ATG7	GAGCAGCCTTGTGAGAGACA	GGATGCACTGGATACCAGCA
LC3B	TATCACCGGGATTTTGGTTG	GAGAAGACCTTCAAGCAGCG
ULK2	CCTGCACAGCAAAGGAATCA	CGAGCAAAACCAAAATCCGCT
ULK1	CCTGCACAGCAAAGGAATCA	CGAGCAAAACCAAAATCCGCT
ACTB	GACCTCTATGCCAACACAGT	AGTACTTGCGCTCAGGAGGA

Table 2. Primary antibodies used in the present study

Antibody	Species	Dilution	Company(Catalog#)
ERas	Rabbit	1:1000	Sigma
Flag	Mouse	1:2000	CST
p62	Rabbit	1:2000	CST
LC3B	Rabbit	1:2000	Sigma

p-Akt	Rabbit	1:2000	CST
Akt	Rabbit	1:2000	CST
p-mTOR	Rabbit	1:2000	CST
mTOR	Rabbit	1:2000	CST
p-ULK1	Rabbit	1:2000	CST
ULK1	Rabbit	1:2000	CST
Caspase3	Rabbit	1:1000	CST
Cleaved-caspase3	Rabbit	1:1000	CST
p-p38	Rabbit	1:2000	CST
p-JNK	Rabbit	1:2000	CST
Actin	Mouse	1:5000	CST
GAPDH	Rabbit	1:5000	CST
