# Supplementary material for: ERas Enhances Resistance to Cisplatin-Induced Apoptosis by Suppressing Autophagy in Gastric Cancer Cell
Source: Front Cell Dev Biol. 2020 Jan 21;7:375. doi: 10.3389/fcell.2019.00375 (PMC7005724; doi:10.3389/fcell.2019.00375)

Figure1

A

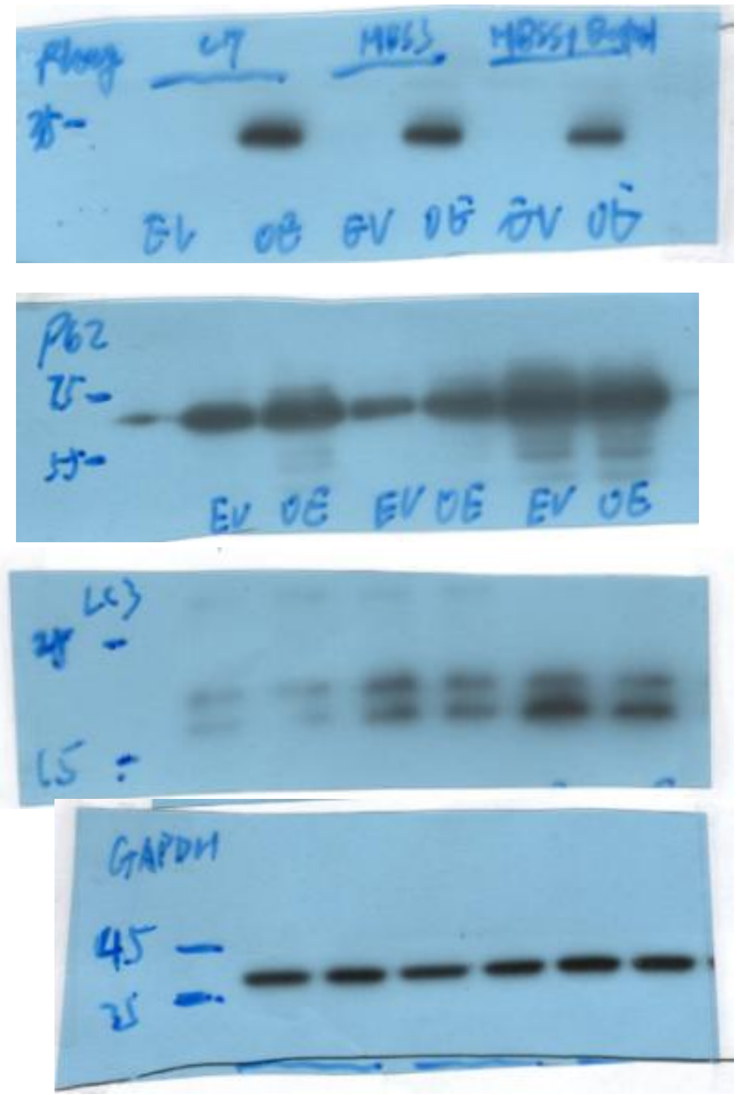

FLAG

P62

LC3B

GAPDH

B

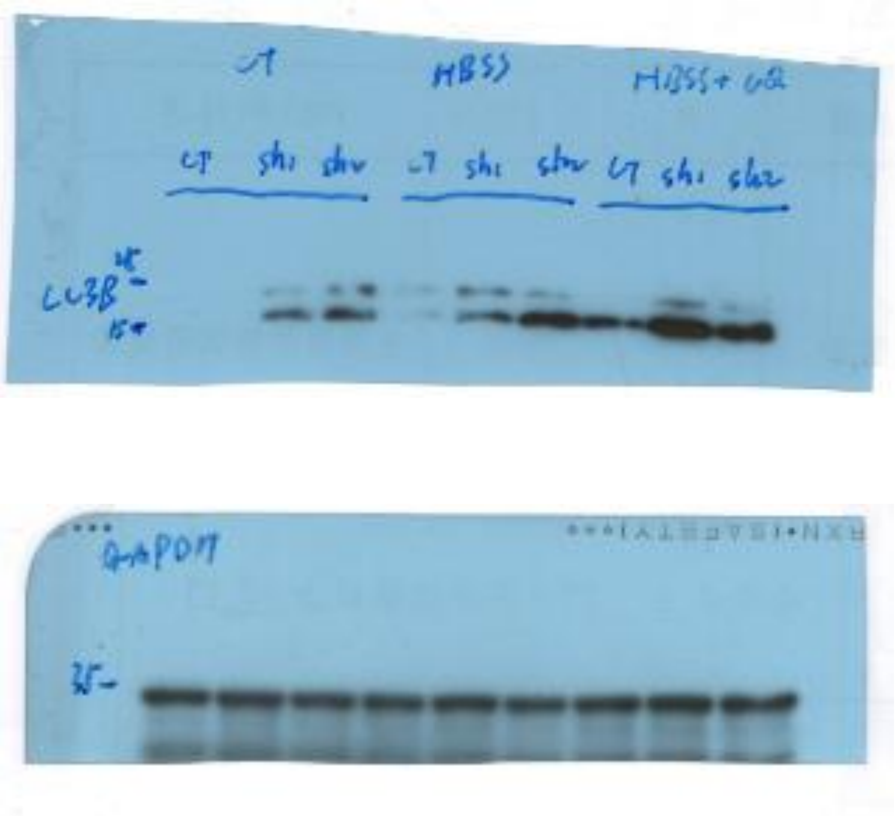

LC3B

GAPDH

Figure3

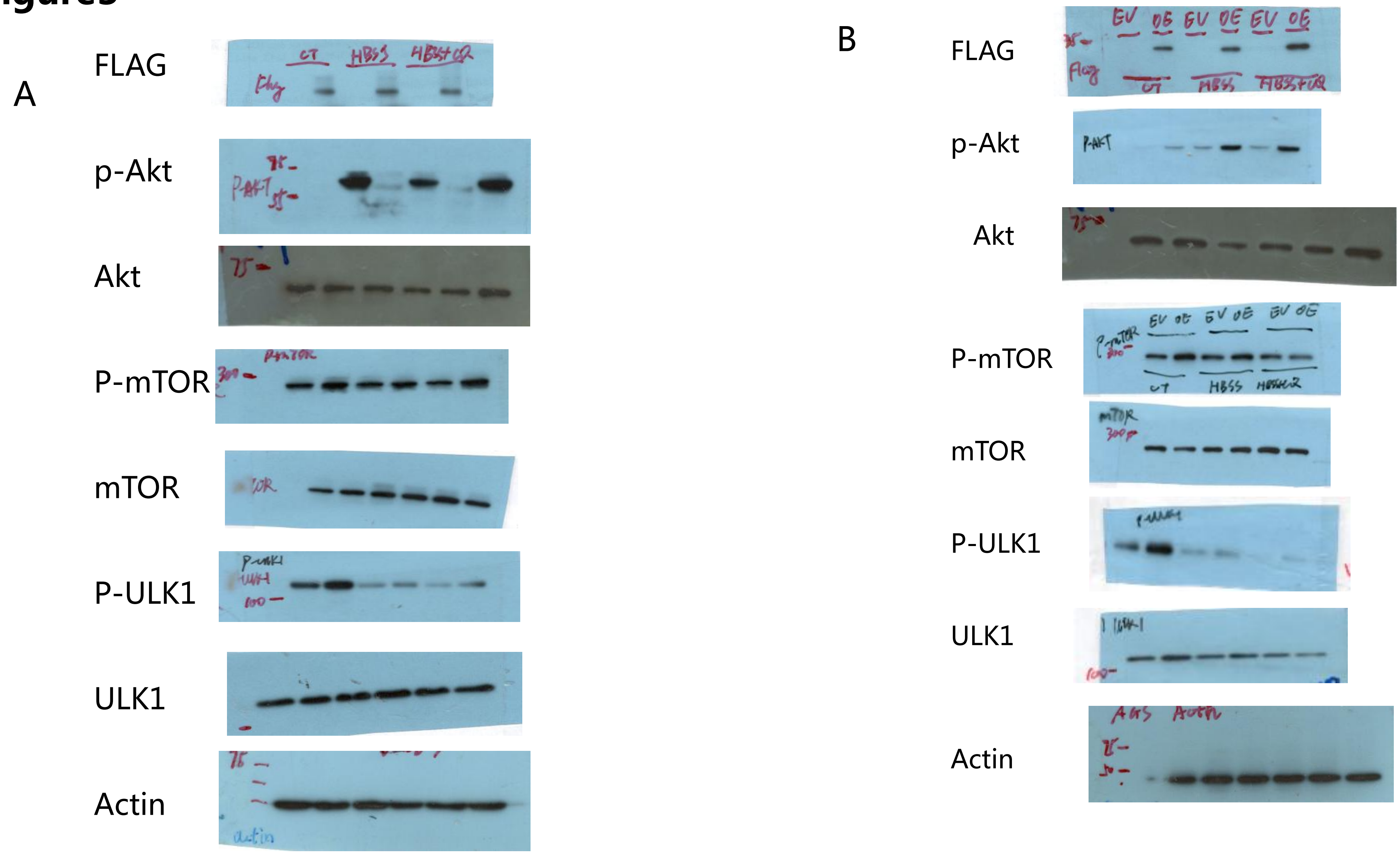
A
FLAG
p-Akt
Akt
P-mTOR
mTOR
P-ULK1
ULK1
Actin
B
FLAG
p-Akt
Akt
P-mTOR
mTOR
P-ULK1
ULK1
Actin

Figure4

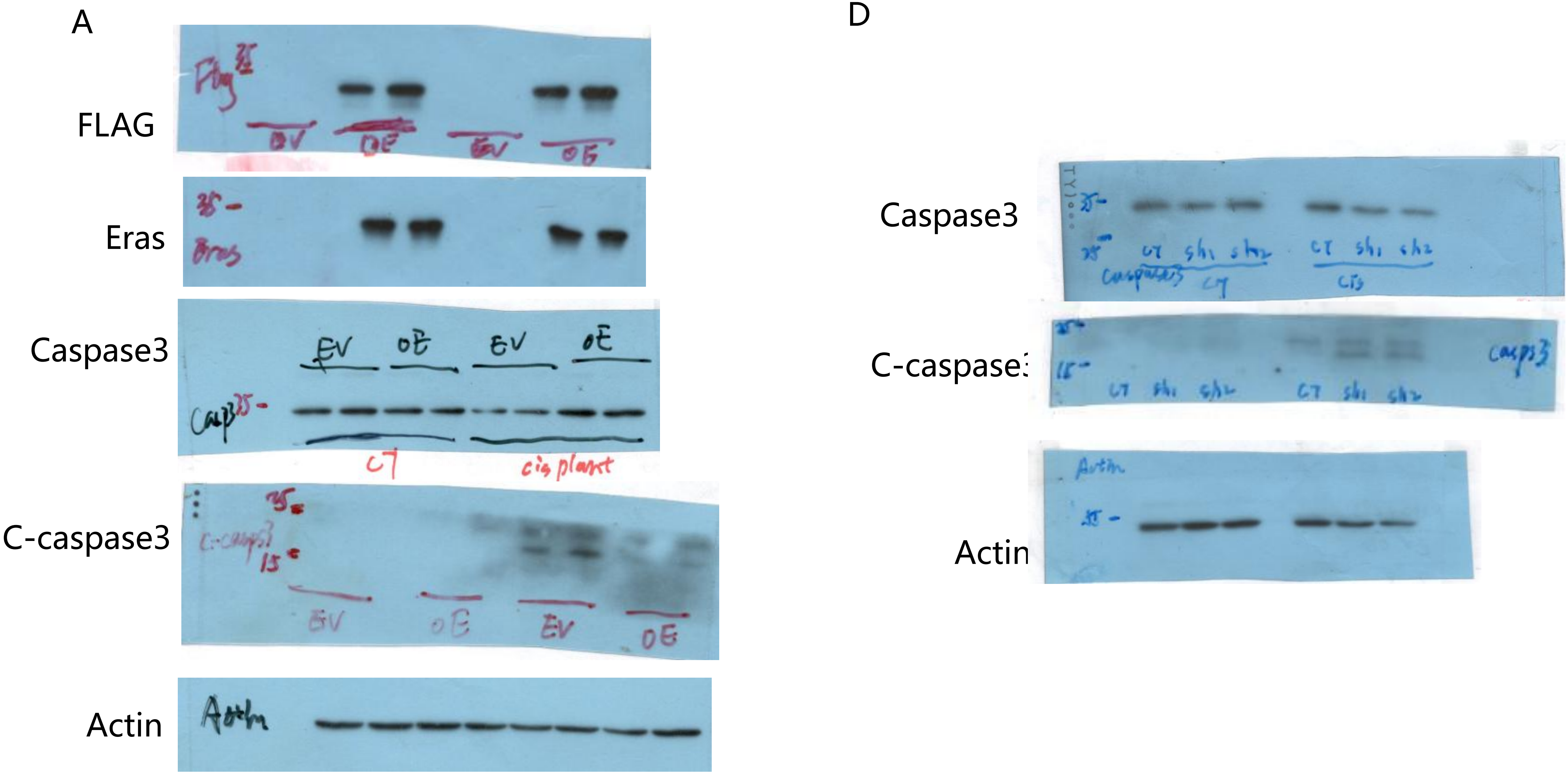
A
FLAG
Eras
Caspase3
C-caspase3
Actin
D
Caspase3
C-caspase
Actin

Figure5

A

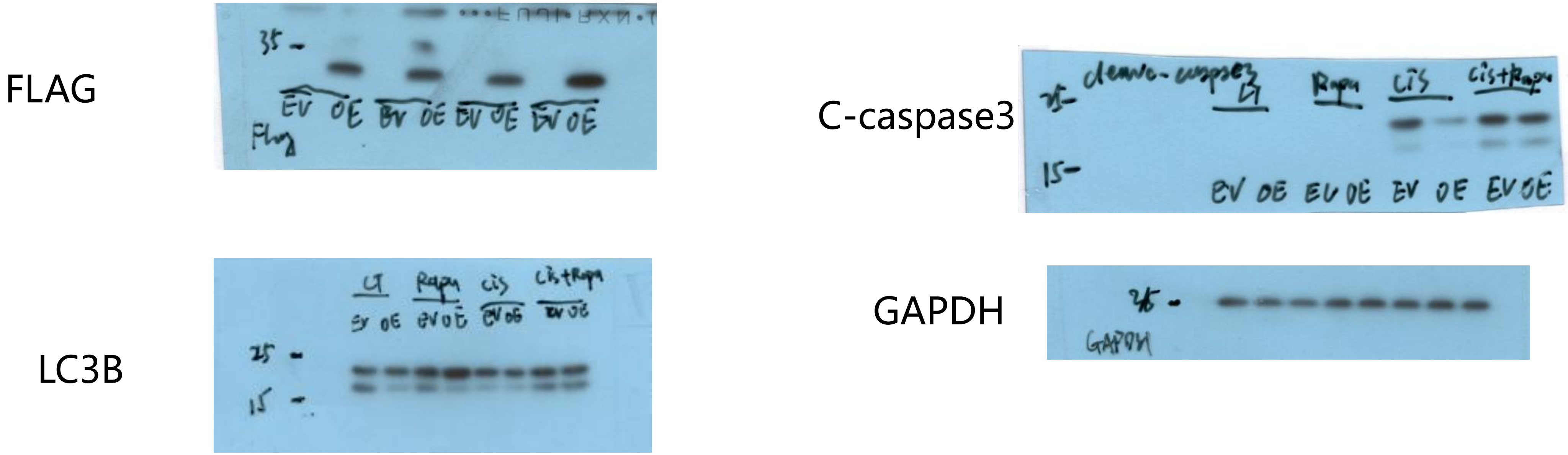

S1

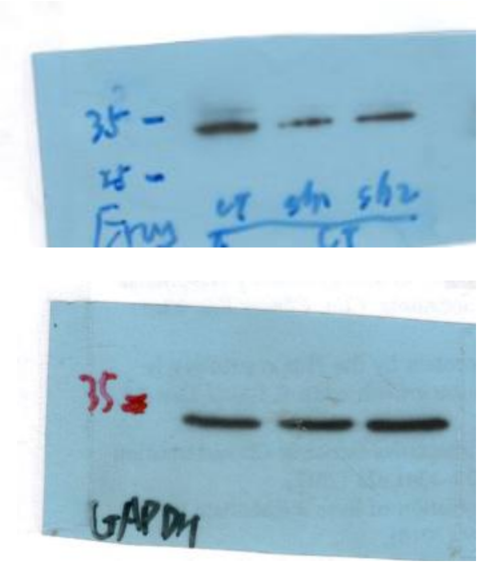

S2

A

FLAG

P62

LC3B

GAPDH

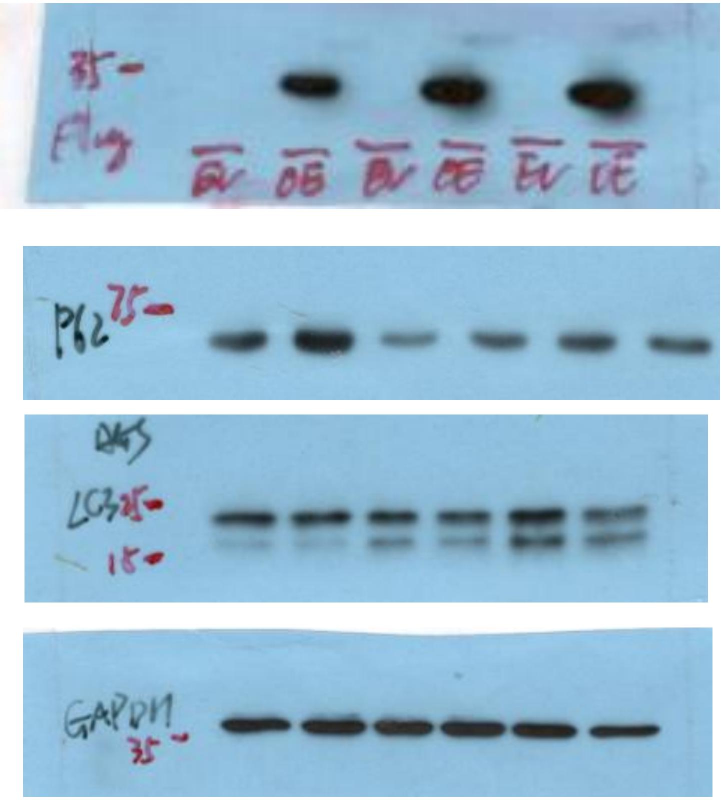

B

LC3B

GAPDH

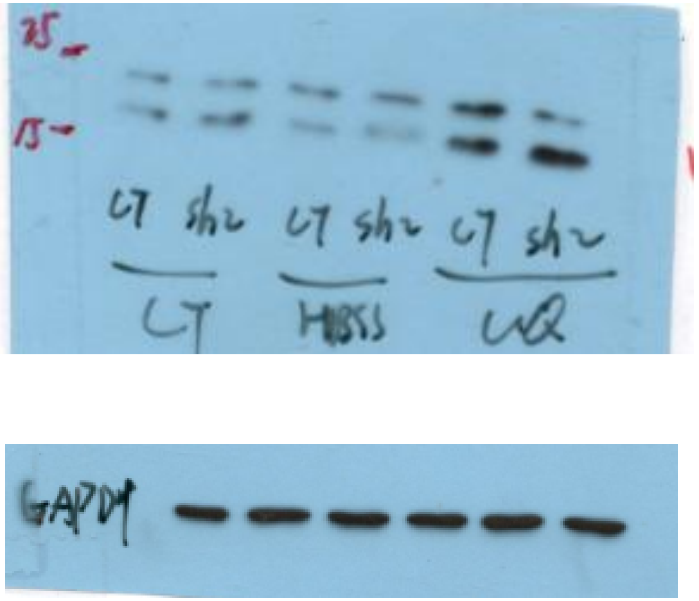

S4

A

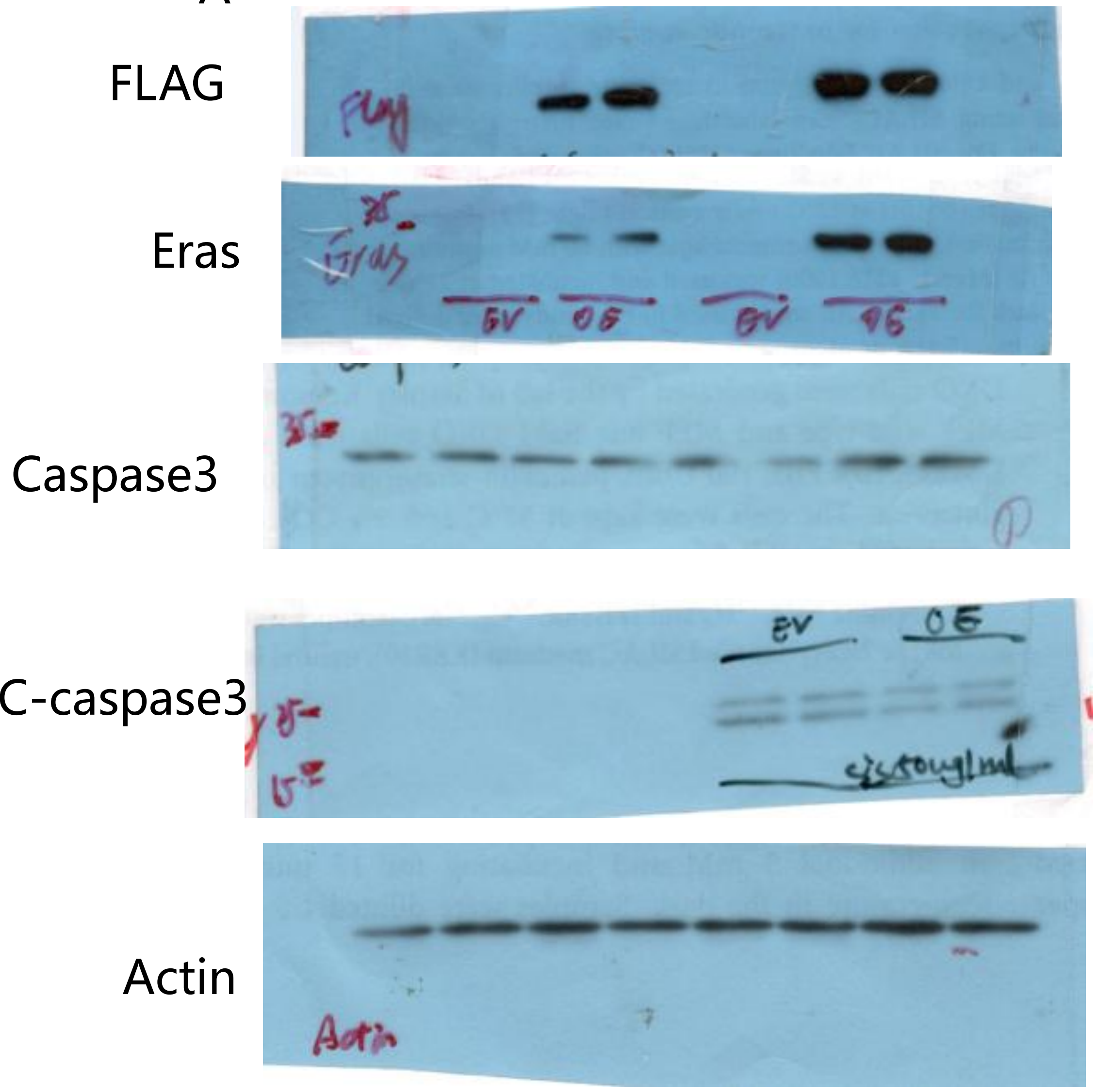
FLAG
Eras
Caspase3
C-caspase3
Actin

D

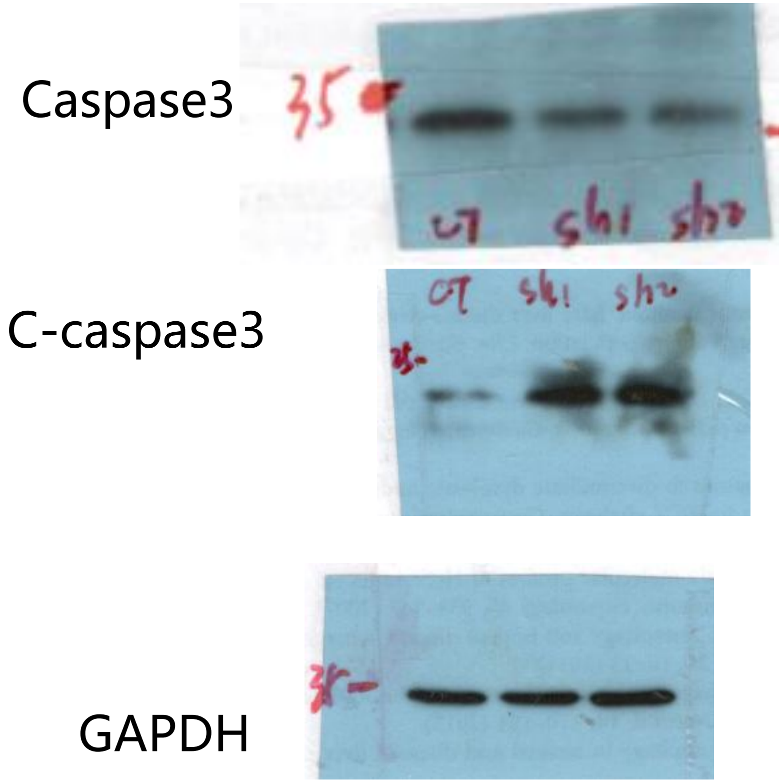
Caspase3
C-caspase3
GAPDH

S5

P-p38

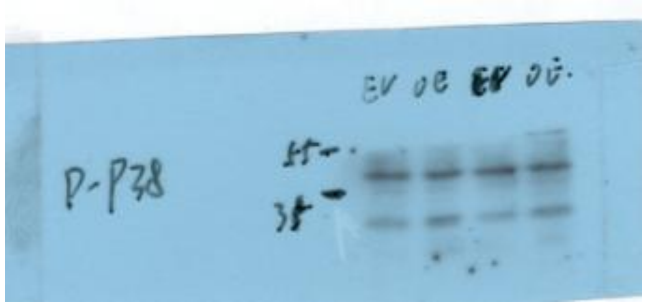

P-JNK

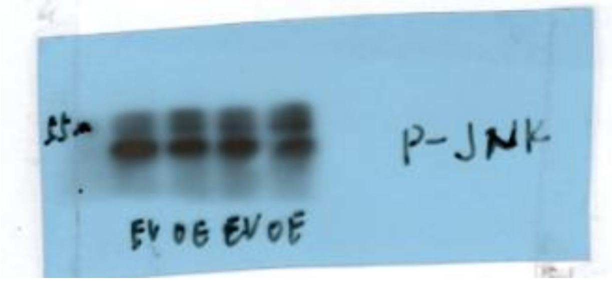

GAPDH

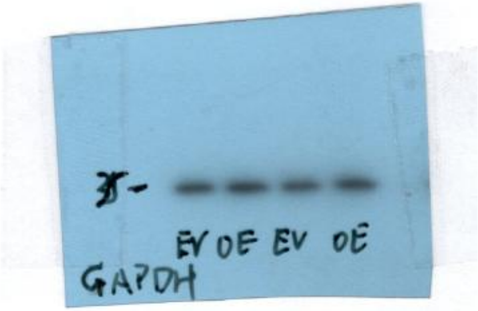

Flag

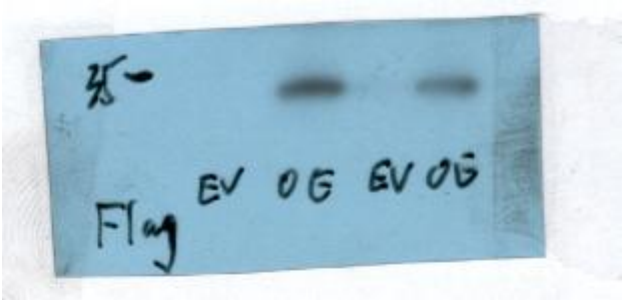

Supplement: DATA SHEET S1 — Raw data. [file Data_Sheet_1.PDF]
